# Euglycemic Diabetic Ketoacidosis With Sodium-Glucose Cotransporter-2 Inhibitor Use Post-Bariatric Surgery: A Brief Review of the Literature

**DOI:** 10.7759/cureus.10878

**Published:** 2020-10-10

**Authors:** Qasim Z Iqbal, Danil Mishiyev, Zeeshan Zia, Raffaele A Ruggiero, Ghulam Aftab

**Affiliations:** 1 Internal Medicine, Northwell Health, New York, USA; 2 Pulmonary Medicine, Saint Peter’s University Hospital, New Brunswick, USA

**Keywords:** euglycemic diabetic ketoacidosis, sglt-2 inhibitors, diabetes mellitus, critical care, critical care and internal medicine education

## Abstract

Sodium-glucose cotransporter-2 (SGLT-2) inhibitors are antihyperglycemic drugs that are currently being recommended as second-line therapy for patients with diabetes mellitus. SGLT-2 Inhibitors function by inhibiting renal cotransporters, which reduces the reabsorption of glucose in the kidney, ultimately decreasing the concentration of glucose in the body. They have gained popularity in recent years due to their protective effects on the heart and kidneys - both organ systems that diabetes mellitus has shown to have a deleterious effect on. However, despite their growing fame, they have been found to increase the risk of euglycemic diabetic ketoacidosis (DKA). Euglycemic DKA is particularly dangerous as there is a chance that it can be missed by clinicians due to glucose levels generally being less than 200 mg/dL. There is an increasing body of literature detailing cases of euglycemic DKA after bariatric surgery. We present a brief review of the literature regarding this important side effect of SGLT-2 inhibitors seen in patients after bariatric surgery.

## Introduction and background

Sodium-glucose cotransporter-2 (SGLT-2) inhibitors are part of a group of drugs that are these days being recommended as second-line therapy for patients with diabetes mellitus. They have gained popularity recently due to both cardioprotective and renoprotective effects. This is especially important considering that these organ systems are severely affected by diabetes mellitus. Unfortunately, despite the growing popularity, they have been found in the literature to increase the risk of euglycemic diabetic ketoacidosis (DKA). We present a brief yet interesting literature review on euglycemic DKA with SGLT-2 inhibitor use seen in patients after they underwent bariatric surgery.

## Review

Our knowledge of diabetes, as well as its complications and how to manage them, is a growing field. One of the relatively new classes of anti-hyperglycemic drugs that have found a lot of attention in recent years are sodium-glucose cotransporter-2 (SGLT-2) inhibitors. SGLT-2 inhibitors work by inhibiting renal cotransporters, thereby reducing the reabsorption of glucose in the kidney and ultimately decreasing the concentration of glucose in the body. Through glucosuria, SGLT-2 inhibitors have been shown to help patients lose weight through the reduction of fat mass in type 2 diabetics [1,2].

Diabetes is known to increase the likelihood of cardiovascular disease as well as the progression of chronic kidney disease to dialysis. SGLT-2 inhibitors have found recent praise for their protective effects on the heart and kidneys [3-5]. The advent has found that the use of some of the drugs in this class has led to significantly improved outcomes, creating a push for the use of SGLT-2 inhibitors as a first-line agent, especially in patients who have type 2 diabetes mellitus with high cardiovascular risk [6,7]. There are, however, risks associated with SGLT-2 inhibitor use. The risks include increased genital and urinary tract infections, fracture, and rarely euglycemic DKA.

Diabetics that meet the criteria for bariatric surgery have also seen some promising results of therapy. Bariatric surgery has shown to decrease mortality in obese patients at 5 years and for up to 10 years after surgery, also showing evidence for decreasing rates of cardiovascular disease and cancer [8]. In some instances, surgery even causes partial or complete remission of diabetes in patients. The benefits of bariatric surgery appear to be caused by a hypocaloric state from the restructuring of the GI tract and the associated weight loss, which does not appear to be reproducible from comparable diet-induced weight loss, signifying a more significant change in insulin sensitivity and metabolic activity [9]. Bariatric surgery is not without its own risks, such as infection, but also has long-term sequelae such as malabsorptive disorders. Although the baseline hyperglycemia decreases in some patients with time, patients may use a variety of oral medications and insulin to control their diabetes post-operatively.

SGLT-2 inhibitors have been used post-operatively in patients who had undergone bariatric surgery [10,11]. One of the risks of SGLT-2 inhibitors, as previously mentioned, is euglycemic DKA. Euglycemic DKA is similar to DKA in that it is usually identified with metabolic acidosis, ketosis, bicarbonate less than 18 mEq/L, and a glucose level that is usually less than 200 mg/dL [12,13]. The difference is that in DKA the blood glucose is usually significantly elevated greater than 250 mg/dL.

This discrepancy makes euglycemic DKA particularly dangerous as there is a chance that it could be missed by clinicians. Usually, similar factors precipitate both euglycemic DKA and DKA. These factors include recent surgery, severe infections, myocardial infarction, stroke, prolonged fasting, and other physical stressors. Unfortunately, much remains uncertain; however, there are theories of mechanisms for euglycemic DKA with SGLT-2 inhibitor use [14].

Euglycemic DKA in surgical patients on SGLT-2 inhibitors is not a new phenomenon. A case series of patients after cardiothoracic surgery showed this phenomenon [15]. The time to onset of in those cases was around 24 hours, and the issue was identified and treated. The literature on euglycemic DKA in patients post-bariatric surgery is growing [11,16-19]. The onset of euglycemic DKA in these cases is often not as rapid as those described in the patients after cardiac surgery. The onset was seen in greater than a week’s time, on average, which would fall past the time of discharge after bariatric surgery for most cases.

Patients that have been found in the literature commonly required to stay in the intensive care after presenting with complaints common to DKA, such as nausea, anorexia, and abdominal pain. These symptoms complicate matters further as those symptoms could potentially be side effects of the surgeries themselves [16]. The management for DKA requires adequate fluid resuscitation, a strict intravenous insulin regiment, close monitoring of the electrolytes, and monitoring of the blood glucose levels, especially in the cases of euglycemic DKA.

The American Academy of Clinical Endocrinologists currently recommends that SGLT-2 inhibitors should be held for at least 24 hours before elective surgery and until patients can tolerate a normal diet [20]. Unfortunately, more specific guidelines do not exist yet, and some cases hold the SGLT-2 inhibitors for much longer before and after surgeries, yet still see some evidence of DKA [15]. There are, however, some specific recommendations for supplementation and dietary changes for patients after bariatric surgery [21]. The initial post-operative diet is progressive from liquids to solids over a period of time that could take several months, and afterward, the amount of caloric intake is physically decreased indefinitely by decreasing the size and absorption of the GI tract [11]. A normal diet hence appears to be up to the clinician's interpretation and clinical judgment.

Diabetes management is a dynamic art, especially in patients after bariatric surgery. The changes in diet, and potentially increasing insulin sensitivity that can lead to remission of type 2 diabetes mellitus, are necessary to prevent hypoglycemia or the prolonged use of drugs that may no longer be directly beneficial. SGLT-2 inhibitors can be found to have merit after bariatric surgery because they do not promote hypoglycemia in patients with significantly changed and decreased caloric intake. Insulin also needs to be continuously adjusted, and a sudden decrease in insulin can lead patients further into a pro-ketotic state, increasing their risk for DKA or euglycemic DKA [11].

Lastly, although the risk of euglycemic DKA remains small, more data need to be gathered to see if SGLT-2 inhibitors do increase the risk of euglycemic DKA. The data could also be put forth toward creating specific guidelines for use of this class of antihyperglycemic drugs in diabetic patients after bariatric surgery. The need for this sort of data analysis is growing with the continuously increasing popularity of SGLT-2 inhibitors.

## Conclusions

The literature has shown a small but significant risk of euglycemic DKA in patients on SGLT-2 inhibitors who undergo bariatric surgery. The SGLT-2 inhibitors are currently popular for their cardiovascular and renal benefits. There is even a debate about whether they could potentially replace metformin as a single primary oral antihyperglycemic agent in patients with these risk factors. Our review emphasizes the importance of more clinical research on these drugs and even more so targeting it at studying prolonged post-operative course of patients on SGLT-2 inhibitors and using it form guidelines for their use after bariatric surgery.
